# A Case of Pregnancy While the Uterus Was External to the Abdomen

**Published:** 1844-01-13

**Authors:** 


### A CASE OF PREGNANCY WHILE THE UTERUS WAS EXTERNAL TO THE ABDOMEN.

BY M. PERFETTI.

A peasant girl, aged fifteen, after a violent effort, found a pain in the loins, which always increased after fatigue. Three years afterwards she perceived one day a round body, which projected from the labia pudendi, but repose and the horizontal position easily effected its reduction. She mentioned it to no one. She was married at twenty, and became pregnant two years afterwards. When she had reached the seventh month she felt great pain, on account of the pressure which the displaced uterus exercised on the neighbouring parts. The term of pregnancy having arrived, and the pains commencing, an ignorant midwife kept her for four days without employing any means for terminating her labour. M. Perfetti being at last called in, and seeing the uterus hanging out between the thighs, dilated the cervix uteri by means of two incisions of about sixteen lines long, one in the anterior lip, the other in the posterior. The accouchement and expulsion of the placenta were then accomplished without difficulty.

When the womb was emptied it was reduced. Then, inflammatory symptoms having disappeared under proper treatment, an elastic gum pessary was placed permanently in the vagina. For the last ten months she had no return of the prolapsus.—*Provin. Med. Journal, from Bulletin delle Scienze Mediche,* and *Gaz. Med.*

